# Testosterone Administration Induces a Red Shift in Democrats

**DOI:** 10.1002/brb3.70651

**Published:** 2025-07-07

**Authors:** Rana Alogaily, Giti Zahedzadeh, Kenneth V. Pyle, Cameron J. Johnson, Paul J. Zak

**Affiliations:** ^1^ Center for Neuroeconomics Studies Claremont Graduate University Claremont California USA; ^2^ Department of Psychiatry Loma Linda University Medical Center Loma Linda California USA

## Abstract

**Objective:**

An experiment was run to test if a testosterone administration would influence the political preferences of 136 healthy males.

**Method:**

Synthetic testosterone or placebo was administered to participants who identified the strength of their political affiliation.

**Results:**

Before the testosterone treatment, we found that weakly affiliated Democrats had 19% higher basal testosterone than those who identified strongly with the party (*p* = 0.015). When weakly affiliated Democrats received additional testosterone, the strength of their party affiliation fell by 12% (*p* = 0.01), and they reported 45% warmer feelings towards Republican candidates for president (*p* < 0.001). Testosterone administration did not affect political preferences for strongly affiliated Democrats or strong or weak Republicans.

**Conclusion:**

Our results demonstrate that testosterone induces a “red shift” among weakly affiliated Democrats, providing evidence that testosterone affects political preferences.

## Graphical abstract

Weakly affiliated Democrats had 19% higher basal testosterone than strongly affiliated Democrats.

Testosterone administration in weakly affiliated Democrats reduced alignment to their party by 12% compared to the same group given a placebo.

Weakly affiliated Democrats who were administered testosterone reported 45% warmer feelings towards Republican candidates compared to those given a placebo.

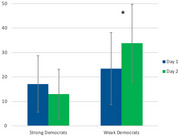

## 1 Introduction

Political preferences are thought to be largely determined by young adulthood (Alwin et al. 1991; Weiss 2020). Political partisanship motivates individuals to vote for the party with which they identify and to interpret political information in ways that are sympathetic to their party's policy stances (Sanders et al. 2002; Bartle and Bellucci 2014). Partisan identities are stable personality features and rarely change due to campaign ephemera (Muirhead and Rosenblum 2020). Party identification has been conceived as an affective attachment to a social group (Green et al. 2004). One's genes appear to explain up to 50% of party affiliation, leaving an opportunity for life experiences to alter political preferences (Hatemi et al. 2010). The brains of conservatives and liberals may even be different (Schreiber 2017), though the evidence for this is mixed (Zmigrod and Tsakiris 2021).

Even with all these indicators, why people vote for or support one political candidate over another rather than simply voting for their own party is poorly understood (Shor and Rogowski 2018; Castle et al. 2017). Several lines of evidence suggest that Democrats are more open to new ideas and cognitively flexible compared to Republicans (Eichmeier and Stenhouse 2019; Haas et al. 2017; Merolla et al. 2013; Caprara et al. 1999). Suggestive research has shown that physical strength and income together reduce support for redistributive policies that align with political conservatives’ views (Petersen et al. 2013). At the same time, research and casual observation have shown that people increasingly ignore information that contradicts their political views, thereby increasing polarization (Huddy et al. 2015; Schreiber et al. 2020).

Even voting can be seen as irrational since the time and effort of casting a vote is unlikely to change the outcome of an election and produce any benefit to oneself (Rogers et al. 2013; Blais and Young 1999). Most studies show that political attitudes and voting behavior are unaffected or only marginally affected by advertising or political debates (Le Pennec and Pons 2019; Coppock et al. 2020; Guess et al. 2021), though social media may matter (Lin 2017). Taken together, this suggests that political preferences are stable aspects of one's personality (Bakker et al. 2021).

The present research hypothesized that manipulating voters' biological states using a neuroactive hormone, testosterone (T), would influence Democrats to support Republican US presidential candidates. This study is part of an emerging research program that uses techniques in neuroscience to understand political behavior (Zmigrod and Tsakiris 2021). We chose to investigate T because its effects on behavior can be substantial. T increases aggression (Batrinos 2012), risk‐taking (Stanton et al. 2011), and punishment of those who violate social norms and engage in other antisocial and selfish behaviors (Ou et al. 2021; Zak et al. 2009). Men with naturally high T levels are more likely to have physical altercations, divorce more often, spend less time with their children, be hypercompetitive, have more sexual partners, face learning disabilities, and lose their jobs more often than men with lower T (Dabbs and Dabbs 2000). Studies in monkeys show that when beta males become alphas, T rises, indicating that T responds to changes in social status (Rose et al. 1975). The same may be true in human males (Wu et al. 2020).

While T is synthesized in the body's periphery, some of it passes into the brain, where it binds to receptors and influences neural activity (Höfer et al. 2013). The striatum, among many other regions of the brain, has a significant density of T receptors (Hermans et al. 2010). This region of the brain is associated with novelty and anticipation of rewards and may be more active in those reading about political opinions (Gozzi et al. 2010). Judgments about candidates’ physical appearances, including markers of T such as musculature (Sinha‐Hikim et al. 2002), jaw size (Verdonck et al. 1999), and hairiness (Mooradian et al. 1987), correlate with voting choices and election outcomes (Ballew and Todorov 2007; Fernández‐Villanueva and Bayarri‐Toscano 2021). Like most neuroactive chemicals, T varies second by second, preparing people for challenges by changing neural activity and associated behaviors. Yet, the moderate variation in endogenous T often yields fragile associations between T and tasks in experiments (O'Carroll 1998). Alternatively, manipulating T pharmacologically produces causal associations with behavior.

The findings for the effect of T on neural activity and behavior, along with our previous research showing that weakly affiliated Democrats' political preferences could be influenced by synthetic oxytocin administration (Merolla et al. 2013), lead us to hypothesize that synthetic T administration would affect the political preferences of weakly affiliated Democrats and not other political groups. We also hypothesized that Republicans would have higher basal T than Democrats, as physical strength among high‐income men diminishes support for redistributive policies (Petersen et al. 2013; Apicella and Cesarini 2011).

## 2 Materials and Methods

### 2.1 General Procedures

The study was run between March and November of 2011. This time period was chosen because the Democratic and Republican primary campaigns for the US presidency made politics and political choices salient through extensive news coverage. In the months prior to the general election, President Obama's reelection was in doubt. Polls in August 2011 showed that President Obama, running as a Democrat, would be defeated by Republican candidate Mitt Romney by two percentage points. At the same time, Romney was tied with Republican Rick Perry and was ahead in the polls of Republicans Ron Paul and Michele Bachmann by two and four points, respectively.

T administration was used in this study because basal T levels in healthy males vary substantially due to a variety of conditions outside of the experimental setting, including exercise, participating in or abstaining from sex, alcohol consumption, and time of day (Dabbs and Dabbs 2000; Brownlee et al. 2005; Diver et al. 2003; Isidori et al. 2005; Sarkola and Eriksson 2003). These effects can be quite subtle. For example, T increased in sports fans of winning teams and decreased in the fans of losing teams (Bernhardt et al. 1998). This variation in T reduces experimental control and adds noise to the data (O'Carroll 1998). In order to determine the causal effects of T on political preferences, the present study administered a onetime dose of synthetic T to eugonodal men. A onetime administration of T using a moderate dose does not induce anger or violence in healthy males, is cleared from the body within 2 days, and is considered safe (Pope et al. 2000; Kouri et al. 1995; Alexander et al. 1998; Bashir et al. 2022; Nadler et al. 2018; Zak et al. 2009).

### 2.2 Participants

A total of 164 eugonadal men volunteered for this study. Foreign nationals who were ineligible to vote in the election were excluded, and three participants were dropped for having basal T that fell outside the normal range, leaving *N* = 136. The average age of participants was 22.3 years (SD = 6.91), and the sample was moderately ethnically diverse, with participants self‐identifying as Caucasian (74%), Asian (11%), Latino (8%), and African American (6%). Participants also identified themselves as Democrats (44.03%), Republicans (8.21%), and Independents (29.10%). The remainder identified as having another affiliation or no party affiliation. Participants arrived at 8:00 p.m. at the laboratory, provided written informed consent before inclusion in the study, and were informed that they might receive synthetic T. After consent, participants were screened for possible contraindications for T administration by a licensed medical doctor (C.J.J.) and had the opportunity to ask questions about the effects of T. Exclusion criteria included significant medical or psychiatric illness, medications that interact with T, and drug or alcohol abuse. No participants were excluded, and no adverse events occurred. Only men were included, as the T preparation we used, Androgel 1% (AbbVie, North Chicago, Il), is FDA‐approved for men only.

### 2.3 Research With Human Subjects

The Institutional Review Board of Claremont Graduate University approved this study (#1387), and there was no deception of any kind.

### 2.4 Blood Draws, Processing, and Analysis

After consent and medical screening, participants had a 20 mL blood draw from an antecubital vein by a qualified phlebotomist to establish basal total T levels. There are several measures of T one can use, but all are highly correlated, so we measured total T. Participants returned to the lab 16 h after T administration following published pharmacokinetics (Swerdloff et al. 2000) for a second blood draw to measure the change in total T. Assays were performed by Yerkes Biomarkers Core (Atlanta, GA) using kits from Diagnostic Systems Laboratories (Webster, TX). Assay CVs were acceptably low (inter‐assay: 1.55% at 3.04 pg/mL, *n* = 2; intra‐assay: 1.60% at 23.87 pg/mL, *n* = 2).

### 2.5 Drug Administration

After the first blood draw, participants were led to a semiprivate room, asked to remove their shirts, and were given a colorless hydroalcoholic gel containing either 10 g of Androgel (55.8% of the sample) or an identical‐appearing inert substance. A 10 g dose is a typical initial amount used for T replacement therapy (US Federal Drug Administration n.d.). The protocol was double‐blind; that is, neither the participants nor the experimenters knew which substance was provided. Participants received application instructions and were observed spreading the gel on their shoulders and upper back following the Androgel package insert.

### 2.6 Political Preferences and Surveys

**FIGURE 1:**
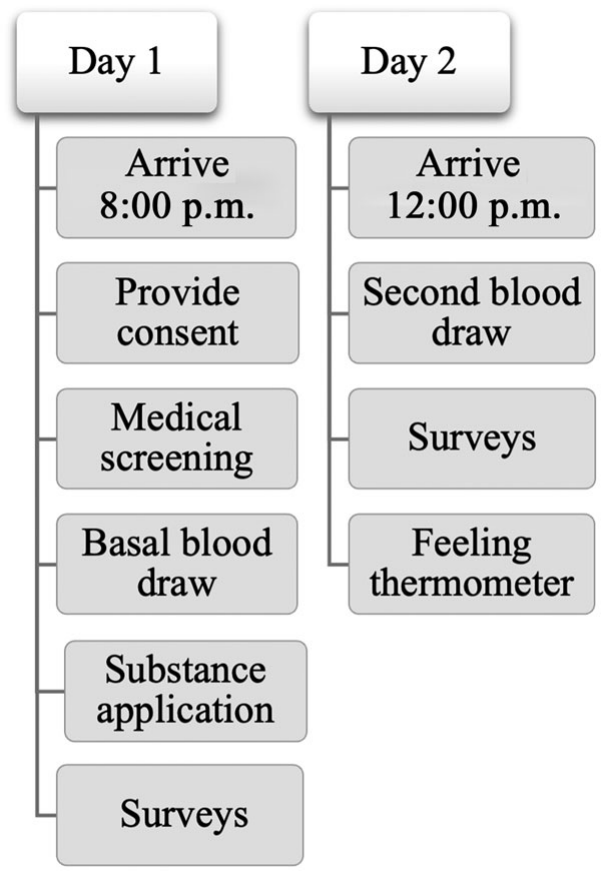
Experiment timeline for Days 1 and 2.

Participants completed questionnaires measuring demographics, trait emotional responses (Affective Intensity Measure [AIM], Larsen, Diener, and Emmons 1986), and an anger inventory (Singer 2007). Political preferences were assessed by ascertaining the strength of party affiliation. Next, a “feeling thermometer” was used to measure support for five Democrats (Barack Obama, Joe Biden, Bill Clinton, Nancy Pelosi, and Harry Reid) and five Republicans (Mitt Romney, Newt Gingrich, Sarah Palin, Mike Huckabee, and Rand Paul) who were running for president or were allied with the sitting president and might run in the future. Feeling thermometers run from 0 to 100 and have been widely used in political science to assess attitudes toward individuals and groups (Wilcox et al. 1989). Participants were asked about their political feelings only on Day 2 in order to avoid framing effects (Tversky and Kahneman 1981). Feeling values were averaged across the five candidates in each party, with higher values indicating greater favorability. Figure 1 shows the timeline of the experiment.

### 2.7 Analytical Approach

The initial analysis tested for within‐subject differences in basal and post‐administration T. These used Student's *t*‐tests and one‐way ANOVAs. These analytical techniques were also used to test if basal T levels differed among political groups and among strength‐of‐affiliation subgroups pretreatment and posttreatment. Next, the effect of T administration on participants' affect and anger was examined for possible influences on political preferences by testing mean differences relative to placebo. Lastly, the impact of T on the strength of political affiliation was tested within political groups by comparing responses for those in the same groups who received a placebo.

## 3 Results

### 3.1 Basal T and Party Identification

There were no differences in basal T levels among the major political parties or independents (Democrats: *M* = 498.86, SD = 185.29; Republicans: *M* = 460.01, SD = 185.41; Independents: *M* = 445.63, SD = 146.93; *F*(4,128) = 0.915, *p *= 0.457). Next, we investigated if basal T varied by strength of party affiliation. Weakly affiliated Democrats had 19% higher average basal T than did strongly affiliated Democrats (Weak: *M* = 529.58, SD = 189.92; Strong: *M* = 445.90, SD = 163.17; one‐tailed *t*(61) = −2.00, *p* = 0.043). No difference in T was found for strongly versus weakly affiliated Republicans (Weak: *M* = 461.47, SD = 253.64; Strong: *M* = 458.78, SD = 131.74; *t*(9) = −0.023, *p* = 0.982). Independents do not have a party, and so were not analyzed for the strength of affiliation.

### 3.2 T Treatment

**FIGURE 2:**
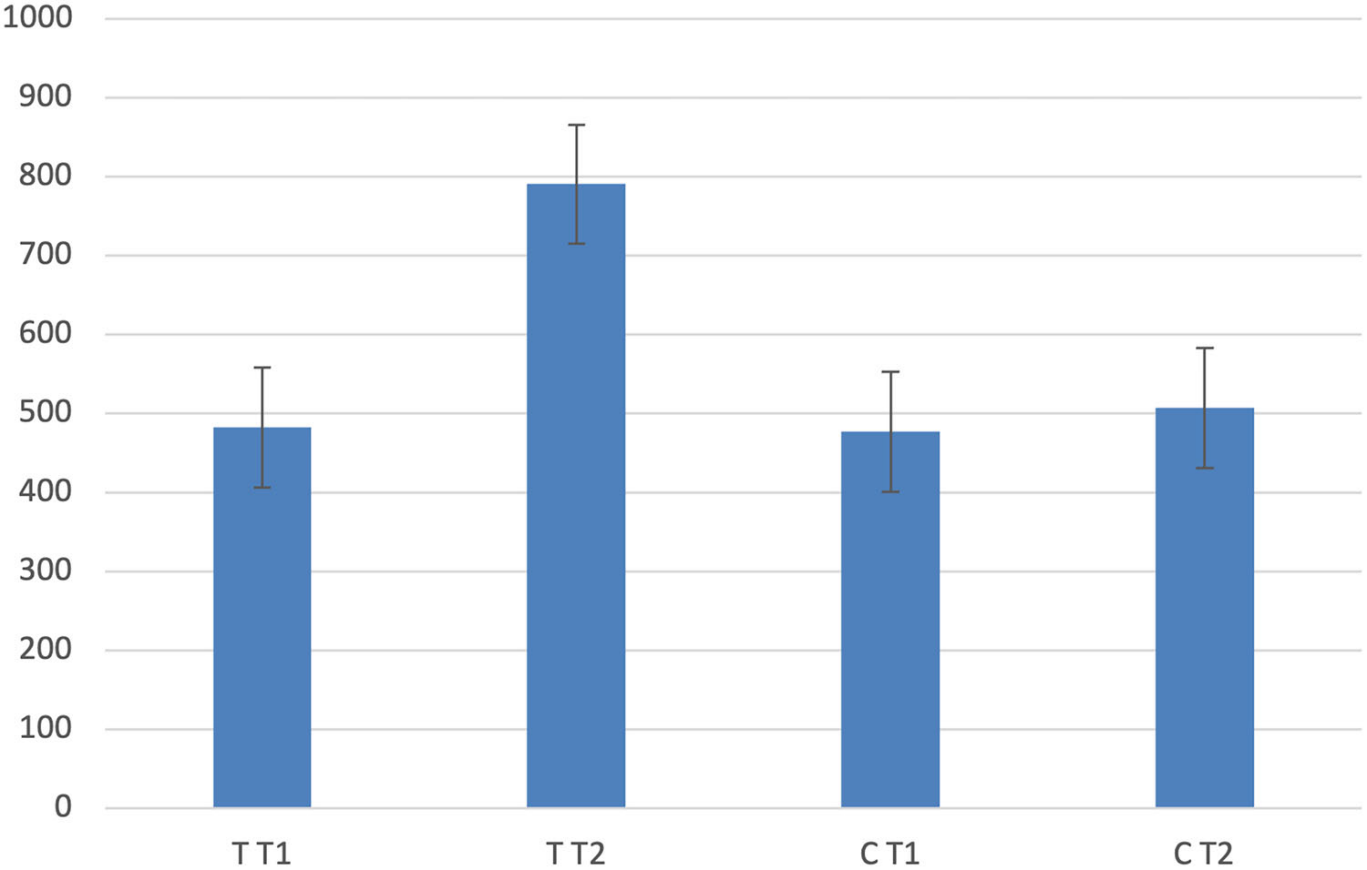
Basal (T1) and posttreatment (T2) average testosterone for the treatment (T) and control (C) conditions. Control average testosterone was unchanged between Days 1 and 2, while the treatment increased average testosterone by 65% (*p* < 0.001). The bars shown are standard errors.

There were 61 participants in the placebo group and 75 in the treatment group. For the placebo group, T levels did not significantly change from before to after substance administration (*M*1 = 478.53, SD1 = 183.17; *M*2 = 495.07, SD2 = 150.65; *t* = −0.999, *p = *0.322). However, average T levels in the treatment group increased by 64.6% (*M*1 = 479.45, SD1 = 161.54; *M*2 = 789.35, SD2 = 230.07; *t* = −12.406, *p *< 0.001; Figure 2). T levels after T administration were identical across party affiliation and for independents (Democrats: *M* = 637.81, SD = 234.57; Republicans: *M* = 610.42, SD = 186.12; Independents: *M* = 634.29, SD = 238.18; *F*(4,128) = 1.316, *p = *0.268).

### 3.3 Treatment and Party Affiliation

**FIGURE 3:**
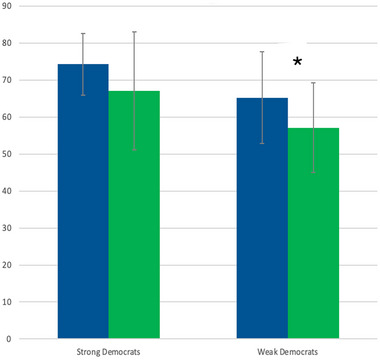
T administration reduced the average strength of party affiliation by 12.5% (*p* = 0.01) for weakly affiliated Democrats but had no effect for strongly affiliated Democrats.

T given to Democrats affected the strength of party affiliation (*F*(1, 63) = 13.94, *p < *0.001). Paired *t*‐tests show the effect was only significant for weakly affiliated Democrats, in whom T administration reduced average party affiliation by 12.5% (*M*1 = 65.25, SD1 = 12.41; *M*2 = 57.12, SD2 = 12.15, *t*(23) = 2.798, *p = *0.01; Figure 3). There was no effect of T on party affiliation for strong or weak Republicans (*M*1 = 66.00, SD1 = 16.25; *M*2 = 62.71, SD2 = 12.97, *t*(3) = 0.943, *p = *0.415).

### 3.4 T and Affect

Average positive affect was unchanged from pretreatment to posttreatment for placebo Democrats (*M*1 = 3.64, SD1 = 0.81; *M*2 = 3.88, SD2 = 0.78; *t*(24) = −1.659, *p* = 0.11) and treatment Democrats (*M*1 = 3.75, SD1 = 0.842; *M*2 = 3.53, SD2 = 0.842; *t*(31) = 1.561, *p = *0.129). Nor did positive affect change for weak Democrats (*M*1 = 3.9, SD1 = 0.852; *M*2 = 3.7, SD2 = 0.733, *t*(19) = 1.165, *p = *0.129). Similarly, positive affect was unchanged in Republicans who received a placebo or T (Placebo: *M*1 = 3.71, SD1 = 0.756; *M*2 = 3.71, SD2 = 0.488; *t*(6) = 0, *p = *1; Treatment: *M*1 = 4.20, SD1 = 0.837; *M*2 = 4.20, SD2 = 0.447; *t*(4) = 0, *p = *1). Positive affect also remained stable for Independents in both conditions (Placebo: *M*1 = 3.50, SD1 = 1.051; *M*2 = 3.60, SD2 = 0.883; *t*(19) = −0.567, *p* = 0.577; Treatment: *M*1 = 3.62, SD1 = 0.973; *M*2 = 3.67, SD2 = 0.730; *t*(20) = −0.271, *p* = 0.789). There were no differences in self‐reported anger due to T treatment (D: *p* = 0.101, R: *p* = 0.810, Ind: *p* = 0.353).

### 3.5 Red Shift

**FIGURE 4:**
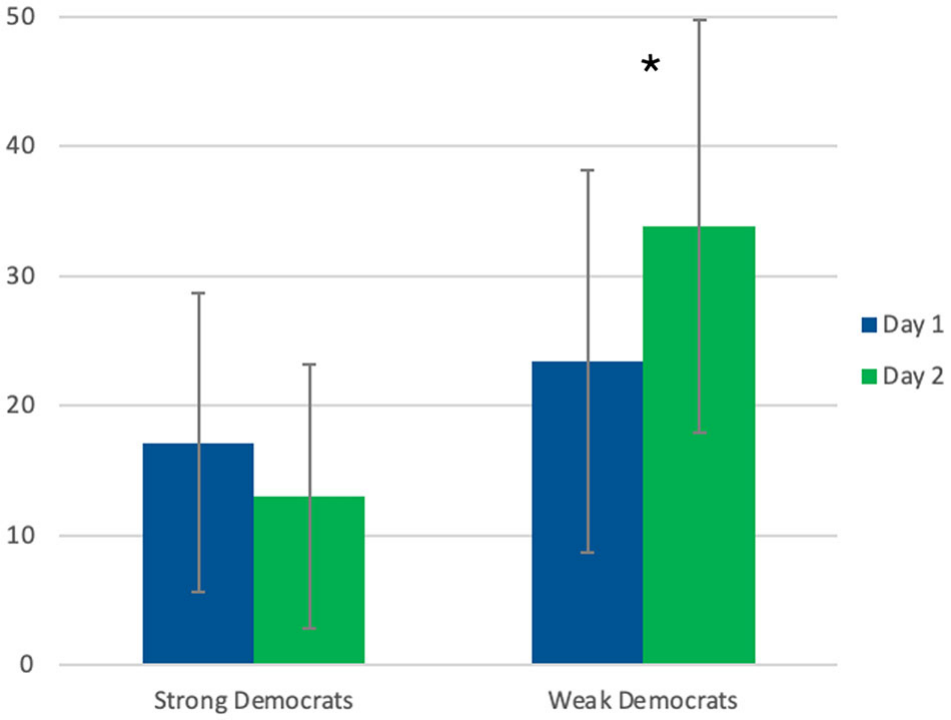
Weakly affiliated Democrats who received testosterone reported a 45% increased average warmth towards leading Republican candidates compared to their baseline average (*p* = 0.014). T did not affect warmth for Republicans by strongly affiliated Democrats.

Warmth by Democrats for Republican candidates increased by 18.2% from Days 1 to 2 using paired *t*‐tests (*M*1 = 23.88, SD1 = 14.431; *M*2 = 28.22, SD2 = 15.072, *t*(58) = −2.018, *p* = 0.048). This effect was not due to a preference change by placebo Democrats (*M*1 = 25.96, SD1 = 14.706; *M*2 = 27.26, SD2 = 12.702, *t*(27) = −0.497, *p* = 0.623). Rather, it was Democrats who received T who produced the change (*M*1 = 22.00, SD1 = 14.152; *M*2 = 29.09, SD2 = 17.097, *t*(30) = −2.140, *p* = 0.041). Comparing Democrats by strength of affiliation, increased warmth towards Republican candidates was driven by weakly affiliated Democrats in the treatment condition (*M*1 = 23.24, SD1 = 14.747; *M*2 = 33.79, SD2 = 15.892, *t*(23) = −2.651, *p* = 0.014) but did not affect strongly affiliated Democrats (*M*1 = 17.14, SD1 = 11.495; *M*2 = 13.01, SD2 = 10.148, *t*(6) = 1.069, *p* = 0.326; Figure 4).

### 3.6 Republicans and Independents

Republicans had no change in warmth for Democrats from Days 1 to 2 (*M*1 = 31.64, SD1 = 22.429; *M*2 = 29.44, SD2 = 27.066, *t*(10) = 0.602, *p* = 0.560), both for those receiving a placebo (*M*1 = 28.57, SD1 = 17.49; *M*2 = 30.71, SD2 = 17.16, *t*(6) = −0.817, *p* = 0.445) and T (*M*1 = 37.00, SD1 = 31.696; *M*2 = 27.20, SD2 = 19.267, *t*(3) = 1.189, *p* = 0.320). Independents who received T showed no change in warmth for Republican candidates (*M*1 = 35.05, SD1 = 14.445; *M*2 = 34.45, SD2 = 15.104, *t*(20) = 0.167, *p* = 0.869) or Democratic ones (*M*1 = 52.05, SD1 = 12.286; *M*2 = 48.18, SD2 = 14.594, *t*(20) = 1.372, *p* = 0.185).

## 4 Discussion

The present study produced three key findings that are mutually consistent. First, weakly affiliated Democrats had basal T levels 19% higher than strong Democrats and all Republicans. Second, the administration of T to weak Democrats reduced the strength of their affiliation with the Democrat party by 13%. Third, weak Democrats who received the T treatment increased their support for Republican presidential candidates by 45%. At the same time, T administration did not influence the political preferences of strongly affiliated Democrats or strong or weak Republicans. The consistency of the effects of T for weakly affiliated Democrats provides confidence that the reported results are due to the treatment rather than chance. Moreover, our results are unlikely to be due to Type 1 errors since our analytical approach systematically went from the broadest comparisons (drug and placebo) to tests for differences in the largest grouping (Democrat compared to Republican) to political subgroups (strong compared to weak affiliations). That is, we did not do a search among all variables for those that happened to show statistical significance and thereby avoided a multiple comparisons problem.

While our experimental design pharmacologically manipulated T in order to demonstrate its causal effect on political preferences, endogenous T changes episodically with a variety of social and environmental conditions, and it is these for which our findings afford insights. One application of the results here would be a novel focus for political messaging. Political campaigns spend hundreds of millions of dollars on political advertising, an amount that increases with every election (Franz and Ridout 2007). Political ads can affect voter turnout (Goldstein and Freedman 2002; Houser et al. 2011 Zak, 2014 and Morris et al., 2019) and may have a short‐term impact on stated candidate preferences (Gerber et al. 2011). A study exploiting media market spillovers found that political advertising affected stated preferences in the 2000 US presidential election using a feeling thermometer and other measures (Huber and Arceneaux 2007). Yet, the consensus view is that the effects of political advertising are small, and this effect is conditional on a large set of variables (Barraza et al., 2015). At the same time, ads with high neurologic Immersion effectively influence behavior (Zak, 2022). Swing voters are key targets of political advertising since they are most likely to be persuaded (Mayer 2007), and weakly affiliated Democrats are the most likely group to vote across party lines (Davis and Mason 2016).

A possible mechanism producing a political preference change after T administration occurs through the action of dopamine. T increases dopamine binding in striatal regions that are associated with risk‐taking (de Souza Silva et al. 2009; de Macks et al. 2011). This could lead Democrats, especially weakly affiliated ones, to take the risk of stating their true preferences for presidential candidates and the strength of their connection to the Democrat party. Weak Democrats would already have been more open to Republican candidates' platform positions compared to strong Democrats, and the additional T appears to have pushed them into a red shift. The self‐report data used here needs to be taken with some skepticism since no actual votes were cast or donations made to Republican candidates. Nevertheless, the neural mechanisms are consistent with previous findings in T administration studies (Zak 2012; Zak et al. 2009).

One clue supporting this reasoning is that weakly affiliated Democrats had a 19% higher average basal T compared to strong Democrats. Weak Democrat males' higher T suggests they were physiologically different than the three other subgroups tested, and this may have made them more susceptible to T administration. For example, in a study of personality traits, Republicans reported a desire for dominance, a trait associated with high T (Sellers et al. 2007; Laustsen et al. 2015). This suggests that weakly affiliated Democrats may be “wolves in sheeps' clothing” and may have consciously or unconsciously stated a Democratic party affiliation when a Republican one would be more appropriate. At the same time, one must be careful not to misinterpret our results: the analysis did not show that Republicans had a higher average T than Democrats. Nor did T turn Democrats into Republicans. Rather, the additional T in weakly affiliated Democrats shifted their preferences towards the right side of the political spectrum.

Our results may also shed light on the rise of right‐leaning politicians in a variety of countries, including the United States, Italy, Canada, Hungary, Czechia, Sweden, Finland, and Croatia, among others (Coi 2024). Increased economic competition due to immigration and inflation is correlated with the rise in populism (Rico and Anduiza 2019), and competition is associated with high T (Dabbs and Dabbs 2000). This relationship suggests a biological mechanism may have contributed to movements to the political right. Relatedly, the increase in populist leaders has been associated with criticism of leftist political parties that emphasize future concerns, such as the environment or climate, rather than present needs that are a source of stress for middle‐ to low‐income males who traditionally voted for left‐leaning parties (Marquardt and Lederer 2022). Stress can increase endogenous T, and our results suggest that this could swing weakly left‐leaning voters towards more right‐leaning populist leaders (Buzogány and Mohamad‐Klotzbach 2021). The rise of populism has also coincided with a secular increase in the number of single and never‐married males in the US and other developed countries (Fry 2023). In the US, for example, the proportion of never‐married adults has increased from 15% in 1960 to 31% in 2021 (Marino 2023). Single men, compared to men with romantic partners, more intensely compete for mates, take more risks, engage in more violent behaviors, and have more sexual partners—all factors that are associated with high T (Arnocky et al. 2018; Wilson and Daly 1985; Haderxhanaj et al. 2014). The increase in single males, along with the red shift we demonstrated, may be another reason for the rise in right‐leaning populist leaders.

A weakness of the study is the underrepresentation of Republicans. This is due to the use of a convenience sample of college students. Future research should examine the effects of T on older adults as well as on women, either by direct administration of T or by using primes, such as videos, known to increase T endogenously. The findings reported here, along with additional research, will help close the gap between political consultants who design strategies so their clients win elections and academic research studying which approaches are most effective in influencing swing voters (Brader, 2005). Our results suggest that political advertising depicting emotional themes that raise T could influence swing voters and perhaps elections.

## Author Contributions

**Rana Alogaily**: writing – original draft; formal analysis. **Giti Zahedzadeh**: investigation; writing – original draft. **Kenneth V. Pyle**: investigation. **Cameron J. Johnson**: supervision. **Paul J. Zak**: conceptualization; investigation; methodology; validation; writing – review and editing; project administration; supervision; resources.

## Ethics Statement

This project was approved by the Institutional Review Board of Claremont Graduate University (#1255) and follows the conditions in the Declaration of Helsinki.

## Conflicts of Interest

The authors declare no conflicts of interest.

## Peer Review

The peer review history for this article is available at https://publons.com/publon/10.1002/brb3.70651

## Data Availability

The data that support the findings of this study are openly available in Open ICPSR at https://www.openicpsr.org/openicpsr/, reference number 155441.
